# Diagnostic Performance of Ultrasound-Based International Ovarian Tumor Analysis Simple Rules and Assessment of Different NEoplasias in the adneXa Model for Predicting Malignancy in Women with Ovarian Tumors: A Prospective Cohort Study

**DOI:** 10.1089/whr.2022.0072

**Published:** 2023-04-27

**Authors:** Neha Rashmi, Sweta Singh, Jasmina Begum, Mukund Namdev Sable

**Affiliations:** ^1^ Department of Obstetrics and Gynecology, All India Institute of Medical Sciences, Bhubaneswar, Odisha, India.; ^2^ Department of Pathology, All India Institute of Medical Sciences, Bhubaneswar, Odisha, India.

**Keywords:** ADNEX model, IOTA, malignancy, simple rules

## Abstract

**Background:**

Comparative performance of various ultrasound models in diagnosing ovarian lesions has not been adequately studied. This study aimed to evaluate the diagnostic performance of the International Ovarian Tumor Analysis (IOTA) simple rules and Assessment of Different NEoplasias in the adneXa (ADNEX) models in women with ovarian lesions.

**Methods:**

Women 18–80 years, with an ovarian lesion planned for surgery were recruited in this prospective observational cohort study. Preoperative risk stratification was done by both IOTA simple rules and the ADNEX model. The diagnostic performance of both models was estimated using histopathology as the gold standard.

**Results:**

A total of 90 women were recruited into the study. The IOTA simple rules were applicable to 77 (85.5%) participants and the ADNEX model on 100% women. Both the simple rules and the ADNEX model had good diagnostic performance. The sensitivity and specificity of the IOTA simple rules for predicting malignancy was 66.6% and 91%, while that of the ADNEXA model was 80% and 94%, respectively. The maximum diagnostic accuracy for prediction of both benign and malignant tumors was obtained when cancer antigen-125 (CA-125) was combined with the IOTA ADNEX model (91.0%), but for Stage I malignancy, the maximum diagnostic accuracy was for ADNEX without CA-125 (91.0%).

**Conclusion:**

Both the IOTA models have a good diagnostic accuracy and are of paramount importance in differentiating benign from malignant tumors and predicting the stage of the malignant disease.

## Introduction

Preoperative prediction of ovarian lesions as benign or malignant, and the stage of the disease in malignancy, is of paramount importance for appropriately managing such women. Not all lesions need a surgery, and not all surgeries should be done by the general gynecologist for optimal patient outcomes. Various prediction models have been proposed over time. The International Ovarian Tumor Analysis (IOTA) group published its consensus statement on the terms, definitions, and measurements to describe the ultrasound features of adnexal tumors.^1^ Subsequently, ultrasound-based predictive models were developed like the Simple Rules model,^2^ Logistic Regression Models,^3,4^ and the Assessment of Different NEoplasias in the adneXa (ADNEX) model.^5^

The simple rules model has been previously found to be easy to use, but was not suitable for all adnexal masses.^2,6^ The ADNEX model has been found to be good at predicting benign versus malignant disease and stage of the malignant disease,^7^ but data is scant regarding the comparative performance of simples rules and ADNEX model.^8–10^

Therefore, this study was conducted with the objective of evaluating the diagnostic performance of the simple rules and ADNEX models in women with ovarian lesions using data from a single tertiary care center, with histopathological diagnosis being the gold standard.

## Materials and Methods

This was a prospective observational cohort study conducted in the department of Obstetrics and Gynaecology in a tertiary teaching hospital in India. Ethical approval from the Institutional Ethics Committee (Reference number-IEC/AIIMS/BBSR/PG Thesis/2019–20/20 dated February 8, 2019) was obtained before the commencement of the study. The study duration was from August 2019 to March 2021.

### Inclusion and exclusion criteria

All consecutive women in the age group of 18–80 years, presenting to the outpatient department, with an ovarian mass or lesion detected on clinical examination, or discovered during previous sonographic examination and planned for surgical management were included in the study. Pregnant women, women with ovarian lesions planned for conservative management, and those unwilling for surgery or for giving consent for the study were excluded. Informed written consent was obtained from the participants before their enrolment into the study.

### Study protocol

After enrolment, a detailed history was obtained and a thorough clinical examination, including a per speculum and a bimanual examination for sexually active women, was performed. Routine preoperative investigations as per standard institute practice were done for all patients. Tumor markers in the form of serum cancer antigen-125 (CA-125) level were obtained for all patients. Other tumor markers were performed as indicated.

In addition, all participants underwent standardized ultrasound examination using Mindray ultrasound machines (Mindray Bio-Medical Electronics Co. Ltd., Shenzhen, China) by IOTA certified gynecological ultrasonographers with more than 10 years of experience (S.S. and J.B.). Transvaginal ultrasonography (TVS) was performed for all women using an intracavitary transducer of frequency 5–7.5 MHz, with patients in supine position with knees bent and feet placed flat on the bed shoulder-width apart, with an elevation under the pelvis. In case of incomplete visualization of mass by TVS and in case of virginal females, transabdominal ultrasonography was performed using a transducer of 2–5 MHz frequency, with the women in supine position. For bilateral ovarian lesions, the lesion having the more complex ultrasound morphology was included for analysis. In case of bilateral lesion with similar morphology, the lesion with the larger diameter was included.

Preoperative risk stratification was done using IOTA simple rules to classify the ovarian lesion as benign (B), malignant (M), or inconclusive (I).^1,2^ The B rules considered were (B1) unilocular cyst; (B2) presence of solid components with largest diameter <7 mm; (B3) presence of acoustic shadows; (B4) smooth multilocular tumor with largest diameter <100 mm; and (B5) no blood flow (color score 1). The M rules considered were (M1) irregular solid tumor; (M2) presence of ascites; (M3) presence of at least 4 papillary structures; (M4) irregular multilocular solid tumor with largest diameter ≥100 mm; and (M5) very strong blood flow (color score 4).

The lesion was classified as benign if one or more B rules were applicable in the absence of M rules. Similarly, the lesion was classified as malignant if one or more M rules were applicable in the absence of B rules. If both M-rules and B-rules were applicable, or if no rules applied, the lesion was deemed as inconclusive.

The IOTA ADNEX model was also applied using online algorithm (https://www.iotagroup.org/iota-models-software/adnex-risk-model) available free of cost at the IOTA website. The ADNEX model consists of three clinical parameters and six ultrasonographical parameters.^5^ The clinical parameters are age (years), type of center of ultrasound examination, and serum CA-125 levels. Ultrasound parameters include the maximal diameter of the lesion in mm, presence of >10 cyst locules, percentage of solid component in the lesion, number of papillary projections, presence of acoustic shadows, and presence of ascites. Preoperative triaging of the ovarian tumor was then done into five categories as (1) benign tumors; (2) borderline tumors; (3) early invasive cancer (Stage I); (4) advanced invasive cancer (Stages II–IV) and; (5) secondary metastatic cancer.

The participants underwent surgical management either in the department of obstetrics and gynecology or the gynecological oncology unit as per the results obtained and the specimen was sent for histopathological examination. Findings of IOTA simple rules and the IOTA ADNEX model were then compared taking histopathology as the gold standard.

### Statistical analysis

Based on previous years registry of ovarian tumors at our institute, a sample size of convenience was taken for the purpose of this study. Data of all participants were collected and entered using Microsoft Excel version 2016. Statistical analysis was done using IBM-SPSS (statistical package for the social sciences) version 23. Descriptive statistics used were mean ± standard deviation for continuous variables, and frequency and percentage for categorical variables. Group comparison for continuously distributed data was done using independent sample “t” test when comparing two groups. Chi-square test was used for group comparison for categorical data. Fisher's Exact test was used if the expected frequency of contingency tables was found to be less than 5 for more than 20% of the cells.

Statistical significance was kept at *p* < 0.05. The diagnostic performance of IOTA simple rules was calculated in terms of sensitivity, specificity, negative predictive value (NPV), positive predictive value (PPV), and diagnostic accuracy. The prediction analysis of IOTA ADNEX model was done by plotting receiver operating characteristic (ROC) curves, and by calculating the sensitivity, specificity, PPV, and NPV. The diagnostic performance of both IOTA simple rules and IOTA ADNEX models was reported with a 95% confidence interval in the analysis.

## Results

**FIG. 1.**
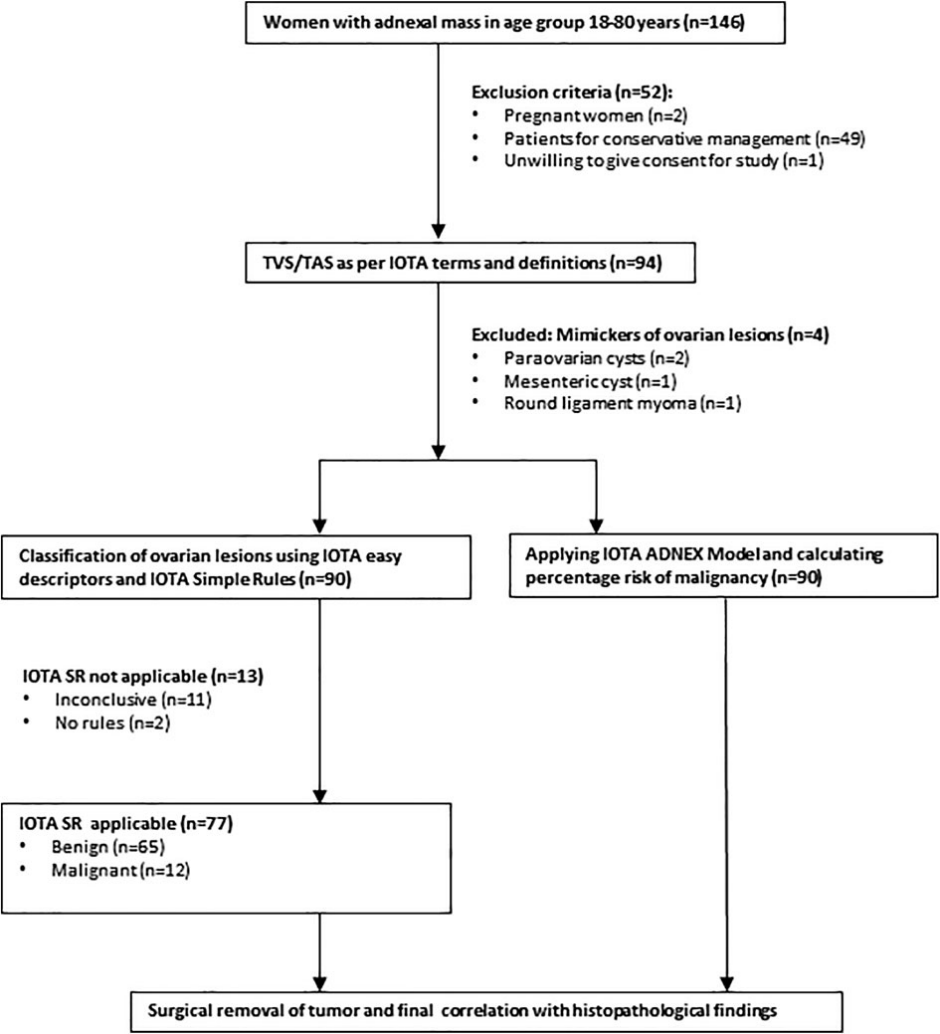
Strengthening the reporting of observational studies in epidemiology (STROBE) flow diagram of the study. ADNEX, Assessment of Different NEoplasias in the adneXa; IOTA, International Ovarian Tumor Analysis; SR, simple rules; TAS, transabdominal ultrasonography; TVS, transvaginal ultrasonography.

There were 146 women with adnexal mass during the study period. Fifty-two were excluded due to various reasons (pregnant women, 2 in number, women already planned for conservative management since no histopathology report would be available for them, 49 in number, and women unwilling to give consent for the study, 1 in number). Thus, 94 women fulfilled the inclusion criteria as per IOTA terms and definitions. There were four cases which were benign as per IOTA simple rules and the IOTA ADNEX model, but with surgical correlation were found to be of nonovarian pathology and were excluded. Thus, a total of 90 participants fulfilling the inclusion criteria were recruited into the study during the study period (Fig. 1; STROBE diagram).

The baseline characteristics of the participants did not show any significant difference between the benign and malignant groups on final histopathology. There was no significant difference in the age between the two groups [benign vs. malignant; 35.4 (12.5) vs. 41.8 (13.4) years]. The CA-125 levels also did not differ significantly between the two groups [benign vs. malignant; 49.7 (81.8) vs. 52.1 (78.3) U/mL]. Other variables like age at menarche, menopausal status, parity, history of tubal sterilization, personal or family history of genital malignancies, body mass index, and history of ovarian cystectomy also did not differ significantly between the women with benign versus malignant disease.

**FIG. 2.**
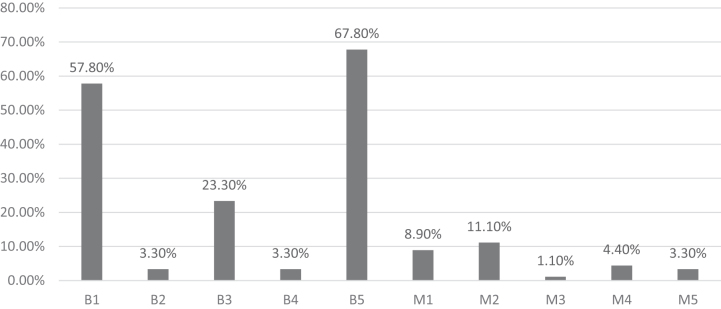
Distribution of individual IOTA Simple Rules-Benign: 65 (72.2%), Malignant: 12 (13.3%), Inconclusives: 13 (14.5%). B, benign; M, malignant.

The IOTA simple rules were applicable to 77/90 (85.5%) participants. Seven women had bilateral lesions. IOTA simple rules could not be applied to 13 participants, as in 11 cases, the ovarian lesions shared features of both M rules and B rules, so result was inconclusive and in 2 cases, the ovarian mass did not share any of 10 characteristics mentioned in IOTA simple rules. Table 1 shows the distribution of the simple rules and descriptors with final histopathological analysis. As per IOTA simple rules, 65 (72.2%) participants had benign tumors, 12 (13.3%) had malignant tumors, and in 13 (14.4%), the result was inconclusive. Figure 2 shows the distribution of individual IOTA simple rules.

**Table 1.** Association of International Ovarian Tumor Analysis Simple Rules and Descriptors with Histopathological Findings

Simple descriptors	Histopathology findings			
	Benign	Borderline	Malignant	Total
Simple ovarian cyst	27	0	0	27
Ovarian dermoid cyst	16	0	2	18
Endometriotic cyst	17	0	0	17
Multilocular ovarian tumor	10	0	1	11
Solid ovarian tumor	2	0	4	6
Solid cystic ovarian tumor	4	0	1	5
Cystic solid ovarian tumor	1	0	1	2
Mucinous ovarian cyst	2	0	0	2
Multilocular cystic solid ovarian tumor	1	1	0	2
Total	80	1	9	90

The commonest benign lesion was B5 (no blood flow) followed by B1 (unilocular cyst), while the commonest malignant lesion was M2 (presence of ascites). Sixty-two of the 65 benign tumors were found to be true negatives and six of the 12 malignant tumors were found to be false positive. Accordingly, the sensitivity of the simple rules for predicting malignancy was 66.6%, specificity was 91%, PPV was 72%, NPV was 95%, and diagnostic accuracy was 88%.

The association of IOTA ADNEX ultrasound features with final histopathological findings is depicted in Table 2. The parameters of maximum tumor diameter (10.1 ± 4.4 cm vs. 14.6 ± 2.7 cm) and presence of solid component (11.2% vs. 66.7%) differed significantly between the histopathologically proven benign and malignant groups.

**Table 2.** Association of International Ovarian Tumor Analysis Assessment of Different NEoplasias in the adneXa Ultrasound Features with Final Histopathological Findings

Ultrasound features	Histopathology findings			*p*
	Benign (*n = 80), n* (%)	Borderline (*n = 1), n* (%)	Malignant (*n = 9), n* (%)	
Maximum tumor diameter (cm)^*^	10.13 ± 4.46	9.95 ± 0	14.61 ± 2.70	0.007
Presence of solid component^*^	9 (11.2)	1 (100.0)	6 (66.7)	<0.001
Size of solid component (cm)	5.28 ± 4.39	3.00 ± 0	9.76 ± 5.27	0.132
Presence of papilla	5 (6.2)	1 (100.0)	1 (11.1)	0.057
Number of papilla				0.167
None	75 (93.8)	1 (100.0)	8 (88.9)	
1 Papilla	5 (6.2)	0 (0.0)	0 (0.0)	
>1 Papilla	0 (0.0)	0 (0.0)	1 (11.1)	
Cyst locules				0.381
≤10	77 (96.2)	1 (100.0)	8 (88.9)	
>10	3 (3.8)	0 (0.0)	1 (11.1)	
Presence of acoustic shadow	19 (23.8)	0 (0.0)	2 (22.2)	1.0
Presence of ascites	10 (12.5)	0 (0.0)	1 (11.1)	1.0

Data expressed as frequency (percentage) with exception of maximum tumor diameter and the size of the solid component, which are expressed as mean (±2 standard deviation).

^*^ Significant at *p* < 0.05.

On applying the IOTA ADNEX model, the true negatives for ADNEX benign and malignant lesions were 74 (82.0%) each and false positives were 6 (7.0%) each. Similarly, the true negatives for ADNEX borderline, early invasive (Stage I), advanced invasive (Stages II–IV), and metastatic disease were 70 (78.0%), 71 (79.0%), 74 (82.0%), and 72 (80.0%), respectively, and the false positives were 10 (11.0%), 9 (10.0%), 6 (7.0%), and 8 (9.0%), respectively. Table 3 shows the actual performance of ADNEX categories. Based on this, the diagnostic performance of IOTA ADNEX model in predicting malignancy is depicted in Table 4. The maximum diagnostic accuracy was obtained when CA-125 was combined with the IOTA ADNEX model for prediction of both benign and malignant tumors (91.0%), but for early invasive malignancy, the maximum diagnostic accuracy was for ADNEX model without CA-125 (91.0%).

**Table 3.** Actual Performance of International Ovarian Tumor Analysis Assessment of Different NEoplasias in the adneXa Categories

Variable	Cutoff	Total positives, *n* (%)	TP, *n* (%)	TN, *n* (%)	FP, *n* (%)	FN, *n* (%)
Histopathology results		10 (11.1)	—	—	—	—
ADNEX benign (%)	≤81.9	14 (15.6)	8 (9)	74 (82)	6 (7)	2 (2)
ADNEX malignant (%)	≥18.1	14 (15.6)	8 (9)	74 (82)	6 (7)	2 (2)
ADNEX borderline (%)	≥6	17 (18.9)	7 (8)	70 (78)	10 (11)	3 (3)
ADNEX early invasive (%)	≥3.7	17 (18.9)	8 (9)	71 (79)	9 (10)	2 (2)
ADNEX advanced invasive (%)	≥1.4	13 (14.4)	7 (8)	74 (82)	6 (7)	3 (3)
ADNEX metastasis (%)	≥1	16 (17.8)	8 (9)	72 (80)	8 (9)	2 (2)

ADNEX, Assessment of Different NEoplasias in the adneXa; FN, false negatives; FP, false positives; TN, true negatives; TP, true positives.

**Table 4.** Comparison of the Diagnostic Performance of Assessment of Different NEoplasias in the adneXa Model in Predicting Malignancy

Predictor	AUROC	95% CI	*p*	Sn, %	Sp, %	PPV, %	NPV, %	DA, %
ADNEX with CA-125: benign (%)	0.800	0.583–1	0.002	80	92	57	97	91
ADNEX with CA-125: malignant (%)	0.800	0.583–1	0.002	80	92	57	97	91
ADNEX with CA-125: borderline (%)	0.723	0.498–0.947	0.023	70	88	41	96	86
ADNEX with CA-125: Stage I (%)	0.819	0.624–1	0.001	80	89	47	97	88
ADNEX with CA-125: Stages II–IV (%)	0.758	0.535–0.981	0.006	70	92	54	96	90
ADNEX with CA-125: metastasis (%)	0.777	0.543–1	0.004	80	90	50	97	89
ADNEX without CA-125: benign (%)	0.786	0.579–0.993	0.003	70	91	50	96	89
ADNEX without CA-125: malignant (%)	0.786	0.579–0.993	0.003	70	91	50	96	89
ADNEX without CA-125: borderline (%)	0.662	0.447–0.877	0.097	70	76	27	95	76
ADNEX without CA-125: Stage I (%)	0.796	0.608–0.984	0.002	70	94	58	96	91
ADNEX without CA-125: Stages II–IV (%)	0.781	0.573–0.988	0.004	70	91	50	96	89
ADNEX without CA-125: metastasis (%)	0.763	0.543–0.982	0.007	70	88	41	96	86

AUROC, area under ROC curve; CA-125, cancer antigen-125; CI, confidence interval; DA, diagnostic accuracy; NPV, negative predictive value; PPV, positive predictive value; ROC, receiver operating characteristic; Sn, sensitivity; Sp, specificity.

**FIG. 3.**
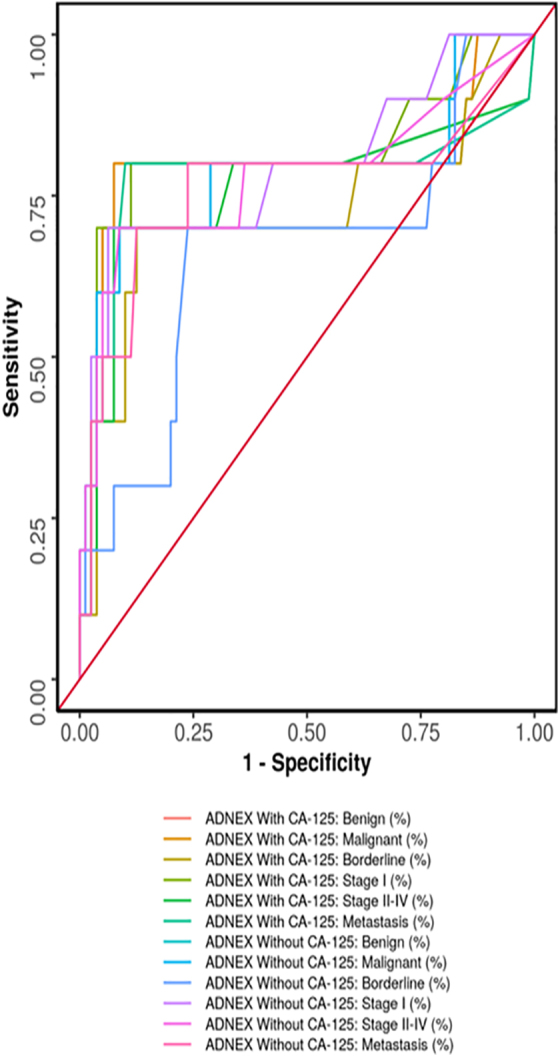
Receiver operating characteristic curve showing diagnostic performance of AXNEX model with and without CA-125. CA-125, cancer antigen-125.

On applying the IOTA ADNEX model on 13 indeterminate lesions by IOTA simple rules, we found that in histopathologically proven benign tumors, the IOTA ADNEX model correctly predicted in >85%, cases and in histopathologically proven malignant tumor, the risk of malignancy obtained by the ADNEX model was 40.4%. The combined ROC curve of the IOTA ADNEX model in predicting malignancy is depicted in Figure 3.

## Discussion

In this prospective observational study conducted on women with ovarian lesions, both the IOTA simple rules (in applicable cases) and the ADNEX model had good diagnostic performance. The sensitivity of the IOTA simple rules for predicting malignancy was 66.6% and that of the ADNEX model with CA-125 was 80%. The specificity of the simple rules was 91% and that of the IOTA ADNEX model with CA-125 was 94%.

The preoperative differentiation between benign and malignant ovarian lesions needs a combined approach, including the patient's medical history, clinical examination, imaging, and tumor markers profile. Ultrasonography offers inherent advantages of being easily available, affordable, and free from radiation hazards. Subjective evaluation of adnexal lesions by ultrasound-based pattern recognition along with Doppler has earlier been shown to differentiate benign from malignant lesions as well as recognize the specific pathology.^11^ Evaluation of ovarian masses by expert sonographers has been shown to be superior to the risk of malignancy index (RMI) or the risk of ovarian malignancy algorithm prediction model.^12,13^

Performed using the standard terminology by IOTA, ultrasound greatly helps to correctly characterize the ovarian lesions and plan proper management. IOTA-based prediction model has been found to be superior to ROMA for predicting the risk of malignancy in both pre- and postmenopausal women.^14^ In previous studies, which were mainly retrospective in nature, the sensitivity of the IOTA simple rules for predicting malignancy has been found to be ranging from 73% to 96%^15,16^ with specificity ranging from 87% to 98%.^16–19^ In our study, which was prospective in nature, the sensitivity of the IOTA simple rules was 66.6% and the specificity was 91%.

In another prospective study, the sensitivity of simple rules was found to be 92.8% and specificity was 92.9%.^10^ Our lower sensitivity may be because we had four cases that turned out to be of nonovarian pathology and also because we did not consider the indeterminate tumors by simple rules as malignant. Six of the 12 malignant tumors by IOTA simple rules were false positives. This is likely as the pretest probability of a woman having a benign disease in our study was high.

The addition of IOTA ADNEX model not only improved the applicability of the model to all lesions (100%) but also increased its sensitivity and specificity (80% and 94%, respectively, in our study). Other authors have reported the sensitivity of the ADNEX model in predicting malignancy as ranging from 79.2% to 98.4% and specificity from 63.6% to 89.6%.^7,20–22^ A recent article compared the IOTA simple rules with the ADNEX model and the RMI and found that both the ADNEX and simple rules models were excellent at characterizing adnexal masses, which were superior to the RMI in Chinese patients.^7^ We found few studies in Indian population on IOTA simple rules model, but they did not utilize the ADNEX model.^23,24^

The maximum diagnostic accuracy for prediction of both benign and malignant tumors was obtained when CA-125 was combined with the IOTA ADNEX model (91.0%), but for Stage I malignancy, the maximum diagnostic accuracy was for ADNEX without CA-125 (91.0%). This may be due to the fact that the rare ovarian mucinous cystadenocarcinoma is usually diagnosed in Stage I of the disease and the CA-125 values are mostly within normal limits. Other researchers have found the area under the ROC curve of the ADNEX model as 0.924, in agreement with our study.^25^ The IOTA models have greater specificity in predicting malignancy, and do not need a magnetic resonance imaging,^22^ which is a big boon in developing regions.

Of late, the Ovarian-Adnexal Reporting and Data System (O-RADS) ultrasound risk stratification and management system, which incorporates IOTA-ADNEX models with pattern recognition, has been used in the Americas to reduce or eliminate ambiguity in ultrasound reporting resulting in a higher probability of accuracy in assigning risk of malignancy to ovarian lesions, and to provide a management recommendation for each risk category. In our study, we have not applied the O-RADS system, as none of the investigators was trained on the O-RADS system.^26^

### Strengths of the study

Our study is a prospective study, thereby minimizing the chances of any missing data, done in a developing country, where data from the ADNEX model is scant. We triaged ovarian tumors by both simple rules and the ADNEX model, thereby establishing comparative analysis between these two models.

### Limitation of the study

The relatively limited cohort of patients decreases the generalizability of our results. We excluded 52 women, of whom 49 were planned for conservative management, as they did not have a histopathological report. Although the ADNEX models can be applied on tumors not planned for surgery, in our study, we have included only those cases where a definite surgical management was planned, with histopathology as the gold standard. The simple rules were also not applicable in a sizeable proportion (14.5%) of tumors in our study.

## Conclusion

Ultrasound-based IOTA models have a good diagnostic accuracy. In developing countries, the IOTA simple rules and the ADNEX model are of paramount importance in differentiating benign from malignant tumors and predicting the stage of the malignant disease and thereby planning appropriate management.

## Abbreviations Used

ADNEX: Assessment of Different NEoplasias in the adneXa
AUROC: area under ROC curve
CA-125: cancer antigen-125
CI: confidence interval
DA: diagnostic accuracy
FN: false negatives
FP: false positives
IOTA: International Ovarian Tumor Analysis
NPV: negative predictive value
O-RADS: Ovarian-Adnexal Reporting and Data System
PPV: positive predictive value
RMI: risk of malignancy index
ROC: receiver operating characteristic
Sn: sensitivity
Sp: specificity
TAS: transabdominal ultrasonography
TN: true negatives
TP: true positives
TVS: transvaginal ultrasonography
